# Age-associated temporal decline in butyrate-producing bacteria plays a key pathogenic role in the onset and progression of neuropathology and memory deficits in 3×Tg-AD mice

**DOI:** 10.1080/19490976.2024.2389319

**Published:** 2024-08-25

**Authors:** Paula M. Chilton, Smita S. Ghare, Benjamin T. Charpentier, Scott A. Myers, Aakarsha V. Rao, Joseph F. Petrosino, Kristi L. Hoffman, John C. Greenwell, Neetu Tyagi, Jyotirmaya Behera, Yali Wang, Lucy J. Sloan, JingWen Zhang, Christopher B. Shields, Gregory E. Cooper, Leila Gobejishvili, Scott R. Whittemore, Craig J. McClain, Shirish S. Barve

**Affiliations:** a Department of Medicine, Division of Gastroenterology, Hepatology & Nutrition, University of Louisville School of Medicine, Louisville, KY, USA; b UofL Alcohol Research Center, University of Louisville School of Medicine, Louisville, KY, USA; c Norton Neuroscience Institute, 4915 Norton Healthcare Blvd, Louisville, KY, USA; d UofL Hepatobiology COBRE, University of Louisville School of Medicine, Louisville, KY, USA; e Department of Anatomical Sciences & Neurobiology, University of Louisville School of Medicine, Louisville, KY, USA; f Alkek Center for Metagenomics and Microbiome Research, Department of Molecular Virology & Microbiology, Baylor College of Medicine, Houston, TX, USA; g Department of Physiology, University of Louisville School of Medicine, Louisville, KY, USA; h Department of Pharmacology & Toxicology, University of Louisville School of Medicine, Louisville, KY, USA; i Department of Neurological Surgery, University of Louisville School of Medicine, Louisville, KY, USA; j Department of Medicine, Robley Rex VA Medical Center, Louisville, KY, USA

**Keywords:** Alzheimer’s disease, gut microbiome, gut dysbiosis, metagenomics, butyrate-producing bacteria, histone acetylation, tau hyperphosphorylation, tributyrin

## Abstract

Alterations in the gut-microbiome-brain axis are increasingly being recognized to be involved in Alzheimer’s disease (AD) pathogenesis. However, the functional consequences of enteric dysbiosis linking gut microbiota and brain pathology in AD progression remain largely undetermined. The present work investigated the causal role of age-associated temporal decline in butyrate-producing bacteria and butyrate in the etiopathogenesis of AD. Longitudinal metagenomics, neuropathological, and memory analyses were performed in the 3×Tg-AD mouse model. Metataxonomic analyses showed a significant temporal decline in the alpha diversity marked by a decrease in butyrate-producing bacterial communities and a concurrent reduction in cecal butyrate production. Inferred metagenomics analysis identified the bacterial acetyl-CoA pathway as the main butyrate synthesis pathway impacted. Concomitantly, there was an age-associated decline in the transcriptionally permissive acetylation of histone 3 at lysines 9 and 14 (H3K9/K14-Ac) in hippocampal neurons. Importantly, these microbiome-gut-brain changes preceded AD-related neuropathology, including oxidative stress, tau hyperphosphorylation, memory deficits, and neuromuscular dysfunction, which manifest by 17–18 months. Initiation of oral administration of tributyrin, a butyrate prodrug, at 6 months of age mitigated the age-related decline in butyrate-producing bacteria, protected the H3K9/K14-Ac status, and attenuated the development of neuropathological and cognitive changes associated with AD pathogenesis. These data causally implicate age-associated decline in butyrate-producing bacteria as a key pathogenic feature of the microbiome-gut-brain axis affecting the onset and progression of AD. Importantly, the regulation of butyrate-producing bacteria and consequent butyrate synthesis could be a significant therapeutic strategy in the prevention and treatment of AD.

## 1. Background

Alzheimer’s disease (AD) is an age-related progressive neurodegenerative disease characterized pathologically by the formation of extracellular senile plaques mainly composed of β-amyloid aggregates and intraneuronal hyperphosphorylated tau neurofibrillary tangles (NFTs) with a gradual decline in cognitive functions.^1^ Alterations in the gut microbiome (dysbiosis) are increasingly recognized to be associated with alterations in the microbiome-gut-brain axis that participate in the etiopathogenesis of AD.^2^ Animal and human studies have shown that enteric dysbiosis, involving compositional changes in the intestinal microbiome, is associated with the development of neuropathological changes and cognitive and memory deficits in AD pathogenesis.^3,4^ Furthermore, fecal microbial transfers from AD patients lead to impairments in adult hippocampal neurogenesis in recipient rats.^5^ However, the functional consequences of enteric dysbiosis affecting the microbial metabolites involved in linking the gut microbiota and brain pathology in AD progression are only beginning to be understood.

With regards to the functional aspects of the gut microbiome and consequent synthesis of microbial metabolites, an important function of the gut bacteria is the fermentation of indigestible dietary fibers leading to the production of short chain fatty acids (SCFAs). Particularly, among the SCFAs produced by the gut microbiome, butyrate has been demonstrated to encompass multiple health benefits including neuroprotective properties that directly or indirectly influence brain functions.^6,7^ Notably, there are also indications that butyrate has the potential to attenuate relevant neuropathological processes underlying AD pathogenesis.^8–10^ Animal and human studies have reported a decrease in the butyrate-producing bacteria in AD;^3,4^ however, these studies were cross-sectional and provided information only about the static abundance of the butyrate-producing microbial taxa associated with AD pathogenesis. Accordingly, the main goal of this study was to investigate longitudinal age-associated changes in butyrate-producing microbial taxa and the concomitant changes in functionality (butyrate synthesis) along with the impacts on AD progression.

Systematic phenotyping and characterization have demonstrated that the 3×Tg-AD mouse model of AD (3×Tg) gradually develops neuropathological changes involving both Aβ-plaques and NFT in an age-related fashion across the lifespan of 4 to 18 months.^11^ This makes the 3×Tg model ideally suitable to examine the development of age-related gut microbial dysbiosis and its pathogenic role in the onset and progression of AD and associated amyloid and tau pathologies. It is noteworthy that information is limited regarding the compositional and functional changes in the gut microbiome prior to the inception of symptomatic AD. Hence, we used the 3×Tg mice to investigate the causal linkage between age-associated gut microbial dysbiosis, especially the loss of butyrate-producing bacteria, and the onset and progression of AD. Moreover, we tested the efficacy of oral supplementation of tributyrin (TB) as a potential intervention strategy to mitigate the effects of gut microbial dysbiosis affecting butyrate-producing microbial communities and consequent development of AD. TB is a naturally occurring triacylglycerol dietary molecule composed of three butyric acid molecules esterified with glycerol that functions as a butyrate prodrug.^12^ TB is chemically stable, but it is rapidly hydrolyzed by pancreatic and gastric lipases to release butyrate. Complete hydrolysis of one mole of TB has the potential to generate three moles of butyric acid.^13–15^ Work performed by us and others have documented the ability to use oral administration of TB to achieve pharmacologically relevant concentrations of butyrate in rodent plasma.^16,17^ Importantly, besides acting as a butyrate prodrug to enhance systemic butyrate concentrations over a sustained period of time, TB exhibits better pharmacokinetic properties with lower toxicity than butyrate.

The present work profiling the gut microbiome by employing 16S rRNA gene sequencing along with inferred metagenomics analysis using PICRUSt2 determined the impact of age-associated temporal changes in the composition and function of butyrate-producing bacteria in relation to the development of neuropathological changes and memory deficits underlying AD disease in the 3×Tg mice. Examination and determination of the specific features of gut microbiota and microbial metabolites affecting the development of AD-related neuropathology and cognitive dysfunction would be relevant for the development of therapeutic strategies targeting the microbiome-gut-brain axis for AD patients.

## 2. Methods

### 2.1. Animals

Homozygous triple transgenic mice modeling early-onset AD, B6;129-Tg(APPSwe,tauP301L)1Lfa Psen1tm1Mpm/Mmjax (3×Tg; RRID:MGI:3720782), developed at the University of California by Frank LaFerla,^18^ were purchased from MMRC. B6129SF2/J mice (B6.129; RRID: IMSR_JAX:101045) were purchased from Jackson Labs and used as non-transgenic (nTg) control mice. All mice were housed in a specific pathogen-free facility with 12 hour light/12 hour dark cycles at the University of Louisville, and experiments were conducted according to federal and institutional guidelines with the approval of the University of Louisville Institutional Animal Care and Use Committee and Institutional Biosafety Committee.

### 2.2. Experimental design

Female 3 × Tg mice more consistently develop age-dependent features of AD-neuropathology involving the accumulation of Aβ plaques and NFTs consisting of phosphorylated tau. Reports have demonstrated consistent, age-dependent, and predictable progression of the AD-like pathology in female, but not male, 3 × Tg mice.^11,19^six-month-old female 3×Tg mice were separated into two groups (*n* = 6 each). One group was treated twice weekly with undiluted tributyrin (TB; Sigma-Aldrich, St. Louis, MO) at 2 g/kg by oral gavage until they were 16 months old, and one group was left untreated. TB dosage was based on our earlier studies that evaluated its role as an effective butyrate prodrug.^16^ At 17 months of age, these mice along with 12 month old (*n* = 6) and 2 month old (*n* = 5) untreated 3×Tg mice were subjected to the behavioral/functional tests described below. When the TB-treated group reached 18 months, mice were deeply anesthetized and perfused with phosphate buffered saline (PBS) after euthanasia by performing a pneumothorax. Liver, cecal contents, fecal pellets, and intestine were collected and the tissue sites clamped off to allow for perfusion with 50 ml 4% formaldehyde (freshly prepared 4% wt/vol from paraformaldehyde in PBS, pH 7.2). Perfused brains were collected, dissected into hemispheres, and fixed overnight in 4% formaldehyde then incubated in 30% sucrose for 72 h at 4°C.

### 2.3. Immunohistochemical staining and immunofluorescence

Brain hemispheres were paraffin embedded, and 5 μm sagittal sections were cut, mounted onto slides, and incubated at 60°C for 48 h. Sections were simultaneously deparaffinized, rehydrated, and antigen unmasked using Trilogy reagent (Sigma-Aldrich, St. Louis, MO) prior to primary antibody staining (Supplementary Table S1), as previously described.^20^ Immunostaining was performed for the detection of neurons, Aβ, tau protein phosphorylated at different sites as well as proteins bearing adducts resulting from oxidative stress processes, 4-hydroxynoneal (4-HNE), and acrolein. Antibody information is listed in Supplementary Table S1. After staining, tissue sections were analyzed primarily in the hippocampus and the subiculum on a Keyence BZ-810 All-in-One fluorescence microscope (RRID:SCR_023617) for both fluorescence and brightfield imaging. Immunofluorescent positive areas were quantified on stained sections using Keyence H3A Analyzer software (RRID:SCR_017375). DAB (3.3’-diaminobenzidine) staining was used for immunohistochemical detection of Aβ, 4-HNE, and acrolein. Aβ positive area was quantified using ImageJ software (RRID:SCR_003070). For 4-HNE and acrolein staining, two forms of quantifications were used, (1) blinded manual counting, and (2) automated quantification of stained puncta. Using the Keyence Analyzer software, parameters were set to detect punctate staining by adjusting both circularity and hue settings to match positive DAB staining. This analysis excluded irregularly shaped areas of matching hues and differentiated between overlapping positive staining events. Both methods yielded similar results, but automated counts from Keyence software are presented. Quantification data are presented average ± standard deviation obtained from 4–6 animals per stain using one tissue section imaging the subiculum per animal.

### 2.4. 16S rRNA gene sequencing

16S rRNA gene sequencing methods were adapted from those developed for the Human and Earth Microbiome Projects.^21^ Briefly, the cecal samples obtained from 3×Tg mice were used to extract total bacterial genomic DNA using the MagAttract PowerSoil Kit (Qiagen, Redwood City, CA). The 16Sv4 region was amplified by PCR and sequenced on the MiSeq platform (Illumina, San Diego, CA; RRID:SCR_016379) using a 2 × 250 bp paired-end protocol, yielding paired-end reads with near complete overlap.^22^ In addition, the sequence reads were demultiplexed, denoised using the Deblur algorithm,^23^ and assigned into operational taxonomic units (OTU) at a similarity of 97% using the latest current SILVA Database^24^ (RRID:SCR_006423) containing only sequences from the v4 region of the 16S rRNA gene to determine taxonomies using usearch70 ‘usearch_global’ function. Biome file was generated for Phylogeny information by aligning the centroid sequences with MAFFT^25^ (RRID:SCR_011811) and created a tree via FastTree^26^ (RRID:SCR_015501). The biome file was summarized, recorded the number of reads per sample, and merged with a file that was generated for the overall read statistics, to produce a final summary file with readings, statistics, and taxonomy information.

### 2.5. PICRUSt2 analysis

Phylogenetic Investigation of Communities by Reconstruction of Unobserved States (PICRUSt2; RRID:SCR_022647) was used for predictive analysis of butyrate-synthesizing pathways in the gut microbiome of 3×Tg mice involved in this study.^27^ Default settings of the q2-PICRUSt2 plugin, which utilizes sequence and gene data information generated in the form of QIIME2 output^28^ with Deblur denoising algorithm. The 16S rRNA gene data were used along with information pertaining to the copy numbers of butyrate-synthesizing genes present within each sequenced archaeal and bacterial taxonomic group in the Integrated Microbial Genomes (IMG) database. The sequence placement with the reference genome database was completed using the SEPP pipeline of the q2-PICRUSt2 plugin. The output file contains normalized values corresponding to the contribution of bacterial genome in a particular sample toward the expression of a specific Kyoto Encyclopedia of Genes and Genomes ID (KEGG Ortholog ID; RRID:SCR_012773).^27^

### 2.6. Animal handling prior to memory and neuromuscular function assessments

Animals were handled for at least 3 days prior to testing to acclimate them the experimental manipulation. Groups of 3×Tg were randomized to blind the testing investigator to treatment or age groups. Tests included novel object recognition test (NORT), Y-maze test, grip test, and rotarod test, described below.

#### 2.6.1. Novel object recognition test

NORT measures the development and retention of recognition memory using the instinctual tendency of rodents to preferentially explore novel objects over known objects.^29^ After a 10-minute training period in an open field containing two similar objects, mice were removed from the area for 4 h and then reintroduced to the area containing an original known object and a novel object and recorded for 5 minutes. The amount of time individual mice spent exploring each object in the field was measured and compared. Results are expressed as the Recognition Index (RI: $\frac{\mathrm{Time}\left(\mathrm{novel}\right)}{\mathrm{Time}\left(\mathrm{novel}\right)+\mathrm{Time}\left(\mathrm{familiar}\right)}$). An RI score of  ≤0.5 indicates memory deficits.

#### 2.6.2. Y-maze test

As a spontaneous alternation test used to assess exploratory behavior and spatial working short-term memory,^30,31^ the Y-maze test also exploits instinctual preference to explore novel areas. Mice were placed in the center of a Y-maze with either three or two open arms, and their movements recorded for 8 minutes. Spontaneous alternation between the 3- and 2-opened arms was assessed, and the percentage of alternation was calculated. Lower scores are indicative of greater functional deficits.

#### 2.6.3. Front limb Grip test

This test was used to assess neuromuscular function as previously published.^32^ Subjects were lifted by the tail to the height where the front paws were the same height as a horizontal bar and then encouraged to grip the bar. Once symmetric, tight grips of both front paws were ensured, the mouse was slowly and gently pulled away until its grasp is broken. The force at which the grip was lost was recorded 3–5 times to determine best performance which is then noted as grip force. Grip strength was calculated as grip force per gram of body weight. Both measurements are directly proportional to neuromuscular function.

#### 2.6.4. Rotarod testing

The rotarod test was used to assess motor coordination and balance. Rotational speed was set to accelerate from 4 to 40 rpm over 300 seconds, and the time (latency) and speed (rpm) required for the animal to fall from the rod were measured. Before starting the rotarod study, all mice were allowed to walk on the rotarod for 1 minute at the constant speed (4 rpm). The speed was then gradually accelerated to 40 rpm in 2 minutes when the rod speed was maintained. A latency time of 300 seconds was recorded for mice that did not fall. The trials were performed four times and were conducted every 15 minutes.^33,34^

### 2.7. Statistics

Excluding 16S rRNA metagenomic and PiCRUSt2 analyses, all statistical analyses were performed using GraphPad Prism 9.2 software (RRID:SCR_002798). Most comparison statistics used ordinary one-way ANOVA using Tukey Post-hoc analysis for multiple comparisons where significance was determined at *p* < 0.05. Parametric unpaired t tests were performed with Welch’s correction, which assumes unequal SD between groups. When data failed normality tests, non-parametric Kruskal-Wallis or Mann-Whitney analyses were performed. For the 16S metagenomics data analysis, the ADONIS function in the R package VEGAN (version 2.4–2; RRID:SCR_011950) was used to perform pairwise comparisons of the community composition between the mouse treatment groups based on the Bray–Curtis dissimilarity matrix (Beta diversity metrics). All pairwise comparisons were significant at *p* < 0.05 using a Benjamini–Hochberg correction for multiple comparisons [http://cran.r-project.org/web/packages/vegan/index.html].

## 3. Results

### 3.1. Age-dependent structural and functional changes in the gut microbiota of 3×Tg mice

To evaluate age-related shifts in the gut microbiome of 3×Tg mice, 16S rDNA sequencing of cecal contents was performed in mice 2, 12, and 18 months of age. A total of 380,989 mapped reads (mean ± SD: 14111 ± 9,804) were obtained from the 28 mice from all groups that were clustered into operational taxonomic units (OTUs) at 97% similarity. The final OTU dataset consisted of 485 OTUs classified into 81 genera, 36 families, 24 orders, 9 classes, and 7 phyla.

#### 3.1.1. An increase in age is associated with a decrease in gut microbial diversity

**Figure 1.**
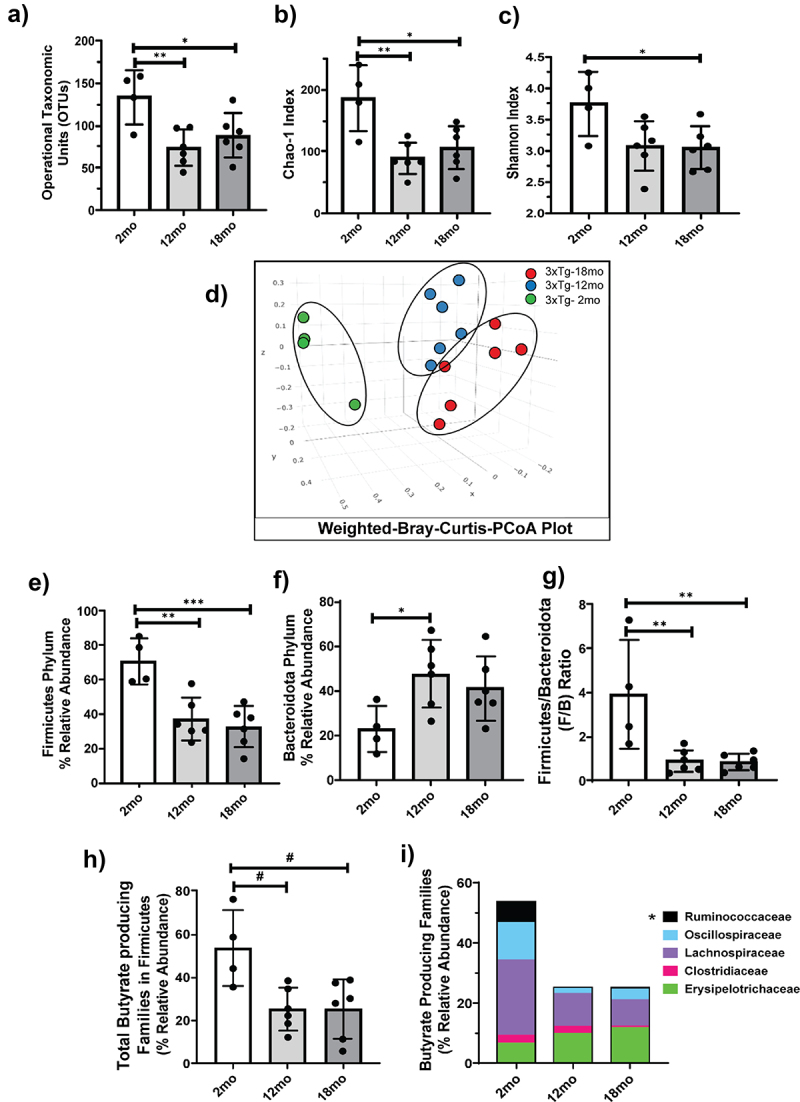
Age-associated decrease in microbial diversity and alterations in microbial composition in 3×Tg mice.

The compositional diversity of the gut microbiome was assessed using both alpha and beta diversity measures. Compared to 2 month animals, 12 month mice exhibited a 50% reduction in sample richness as measured by observed OTU and Chao-1 (both *p* < 0.001; Figure 1a,b), both measures of alpha diversity. These reductions persisted at 18 months (*p* < 0.05). Sample evenness, another component of alpha diversity, was also assessed using the Shannon Diversity Index (Figure 1c). Although reductions in sample evenness were evident in 12 and 18 month mice, only the latter was significantly different from 2 month. These data indicate that alpha diversity of the cecal microbiome significantly decreases with advancing age in 3×Tg mice. In contrast to the 3×Tg mice, nTg mice did not exhibit any age-associated decreases in the gut microbial alpha diversity measures at 12 and 17 months in comparison to 5 months; however, a modest decline in microbial diversity at 17 months, in comparison to 12 months, was noted (Supplementary Figure S1A).

Age-related changes in alpha diversity metrics OTUs a) and Chao1 Index b) measuring species richness and the Shannon Index c) measuring species abundance and evenness present in the cecal contents of indicated groups of 3×Tg mice are shown. Beta diversity PCoA plots d) depicting compositional differences were generated using Weighted Bray–Curtis distances for communities within the microbiome of 2, 12, and 18 month old 3×Tg mice. All pairwise comparisons for Beta diversity were calculated using ADONIS (R vegan package) and are significant at 0.05 using a Benjamani–Hochberg correction for multiple comparisons. Age-associated changes in relative abundance of Firmicutes phylum e), Bacteroidota phylum f), as well as the Firmicutes to Bacteroidota (F/B) ratio are shown g). The relative abundance of butyrate-producing communities from the Firmicutes phylum is shown for total h) and indicated bacterial families in a stacked bar graph i). Data are represented as mean ± SD (*n* = 4–6). Significance analyzed using one-way ANOVA with Tukey’s corrected measures are denoted as *p < 0.05, **p < 0.01, and ***p < 0.001. Student’s *t*-test statistical analyses are denoted as ‘#’ for *p* < 0.05.

Beta diversity, a measure of inter-sample diversity, was assessed using the weighted Bray Curtis distance metric and visualized by principal coordinates analysis (PCoA). Three dimensional PCoA plots demonstrated distinct clustering of the gut microbiota according to age and that the microbial populations were significantly different from one another (*p* < 0.05). In particular, the gut microbiome from 12 and 18 month old mice showed distinct segregation from those in the 2 month group (Figure 1d), indicating that the change in the overarching composition of the gut microbiome occurred within the first 12 months in 3×Tg mice and then largely stabilized.

#### 3.1.2. An increase in age is associated with changes in gut microbiome composition (dysbiosis)

Seven phyla were detected in the cecal contents of our mice. Among these, the two major bacterial phyla, Firmicutes and Bacteroidota, showed significant age-dependent changes in their relative abundance (Supplementary Figure S2A). Specifically, the relative abundance of Firmicutes decreased by 50% in 12 month animals compared to 2 month (Figure 1e). In contrast, the relative abundance of Bacteroidota increased by nearly the same amount over the same period (Figure 1f). Consistent with measures of microbial diversity, both shifts were maintained in 18 month old animals (Figure 1e). Importantly, the Firmicutes to Bacteroidota ratio (F/B), a known indicator for gut microbiome health, was significantly decreased in both 12 and 18 month old mice as compared to 2 months (Figure 1g).

#### 3.1.3. Age-associated gut dysbiosis is marked by a decrease in butyrate-producing bacteria in 3×Tg mice

Preclinical and clinical studies suggest that short-chain fatty acids produced by the gut microbiome play a role in protecting against AD pathology.^10,35,36^ Since we observed an age-dependent decrease in phylum Firmicutes, which harbors the majority of butyrate-producing microbes,^37^ we further examined the effect of age on the relative abundance of butyrate-producing bacteria within this phylum in 3×Tg cecal contents. Based on the catalog of butyrate-producing bacteria created by Singhal et al.,^38^ we identified five butyrate-producing families belonging to the Firmicutes phylum. As with our analysis of Firmicutes as a whole, there was a significant decrease in total butyrate-producing families in 12 and 18 month old compared to 2 month old mice (Figure 1h). Specifically, our data demonstrated that advanced age was associated with a significant decrease in the Ruminococcaceae family, one of the largest butyrate-producing bacterial families (Figure 1i), and a consequent reduction in cecal butyrate levels (Supplementary Figure S2D).

Commensurate with the unaffected microbial diversity, nTg mice neither demonstrated an age-dependent decrease in the phylum Firmicutes nor in the relative abundance of total butyrate-producing bacteria within this phylum (Supplementary Figure S1B,C).

### 3.2. Oral administration of tributyrin mitigates age-associated structural and functional changes in the gut microbiota of 3×Tg mice

Based on the observed age-dependent loss of key butyrate-producing microbes and accompanying decrease in cecal butyrate levels, we examined the therapeutic effects of oral administration of TB. Beginning at 6 months of age, 3×Tg mice demonstrate Aβ protein deposition and initiation of AD pathology.^11,18^ As shown in Supplementary Figure S3A, Aβ accumulation was readily detectable in the subiculum of 7 month old 3×Tg mice. Therefore, oral administration of TB was initiated in 6 month old 3×Tg mice and continued until 16 months of age. Cecal samples were analyzed to assess the impact of long-term TB supplementation on gut microbial dysbiosis and the attendant loss of butyrate-producing bacteria.

#### 3.2.1. TB attenuates the age-associated decrease in gut microbiome diversity and dysbiosis in 3×Tg mice

**Figure 2.**
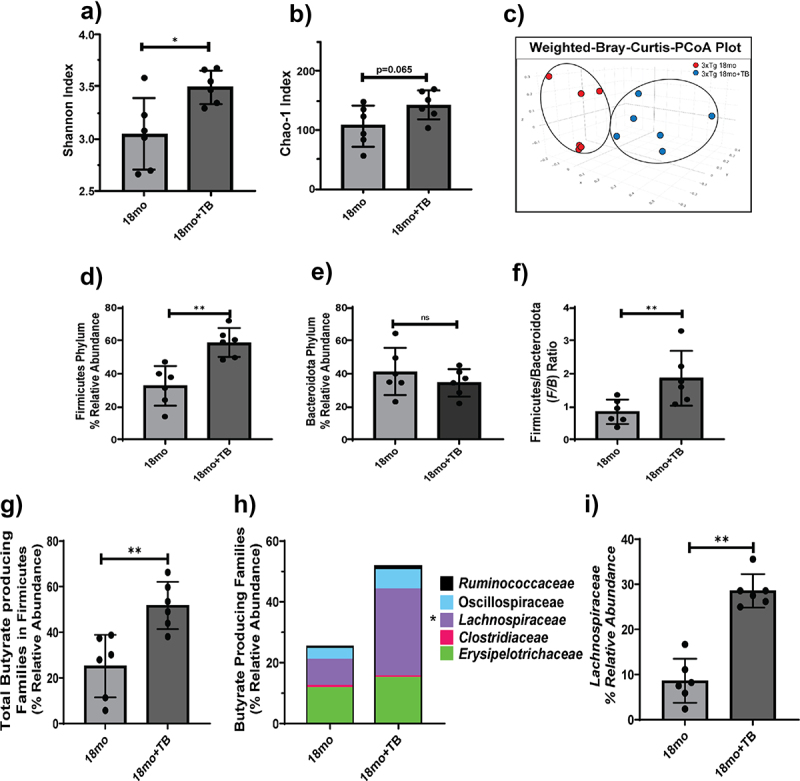
Oral administration of TB prevents the decrease in microbial diversity and composition in 18 month old 3×Tg mice.

Cecal contents of 3×Tg mice given oral TB exhibited a higher degree of microbial evenness as measured by the Shannon Diversity Index than untreated controls (*p* < 0.05; Figure 2a). Furthermore, the richness of the microbiome, assessed by Chao-1 index, also trended higher with TB treatment (*p* < 0.06; Figure 2b). Consistent with alpha diversity measures, beta diversity as visualized using weighted Bray-Curtis PCoA plots (Figure 2c) showed that the gut microbiome from animals undergoing TB treatment were compositionally distinct than from untreated animals. Interestingly, the gut microbiome from TB-administered mice demonstrated less dispersion in their clustering and appeared more similar to the 2 month age group, particularly along the PC1 axis, which indicated TB administration helped maintain a gut microbiome composition similar to that of young mice (Supplementary Figure S2B).

Alpha diversity metrics Shannon Index a) and Chao-1 Index b) as well as Beta diversity PCoA plots c) are shown for 18 month old 3×Tg mice with and without TB treatment. The Beta diversity data are significant at *p* < 0.05, calculated using ADONIS (R vegan package). The relative abundance of communities from Firmicutes d), or Bacteroidota e) phyla, and the F/B ratio f) are shown. Butyrate-producing communities from the Firmicutes phylum are shown as total g) or indicated families in stacked bars h). The relative abundance of the *Lachnospiraceae* family, which harbors the most butyrate producers, is significantly increased in 18 month old 3×Tg given TB compared to no TB treatment i). Data are represented as mean ± SD (*n* = 4–6). Student’s *t*-test statistical analysis was performed, and significance denoted as ns: not significant, *p < 0.05, and **p < 0.01.

Furthermore, taxonomy-based analysis demonstrated that TB also induced changes in the overall distribution of microbial phyla (Supplementary Figure S2C). Specifically, the relative abundance of phylum Firmicutes was nearly 2-fold higher in TB-treated mice as compared to their untreated counterparts (Figure 2d). Without a significant change in phylum *Bacteroidota*, the F/B ratio was significantly greater in mice given TB (Figure 2e,f). Taken together, these data suggest that oral administration of TB prevented changes in the gut microbiome diversity and dysbiosis that occurred in 18 month old 3×Tg mice.

#### 3.2.2. TB attenuates age-associated loss in butyrate-producing gut bacterial families

Oral TB treatment helped to maintain a significantly higher relative abundance of total butyrate-producing families compared to untreated mice (Figure 2g). Specifically, TB led to significant enrichment of bacteria in the major butyrate-producing family, *Lachnospiraceae*, and a marginal increase in *Ruminococcaceae* communities (Figure 2h,i). Furthermore, commensurate with oral TB supplementation, cecal butyrate levels were significantly increased in 18 month old 3×Tg mice even though treatment ended at 16 months of age (Supplementary Figure S2D).

### 3.3. Predicted functional profiles of butyrate synthesizing genes affected by age and tributyrin in the cecal contents of 3×Tg mice

**Figure 3.**
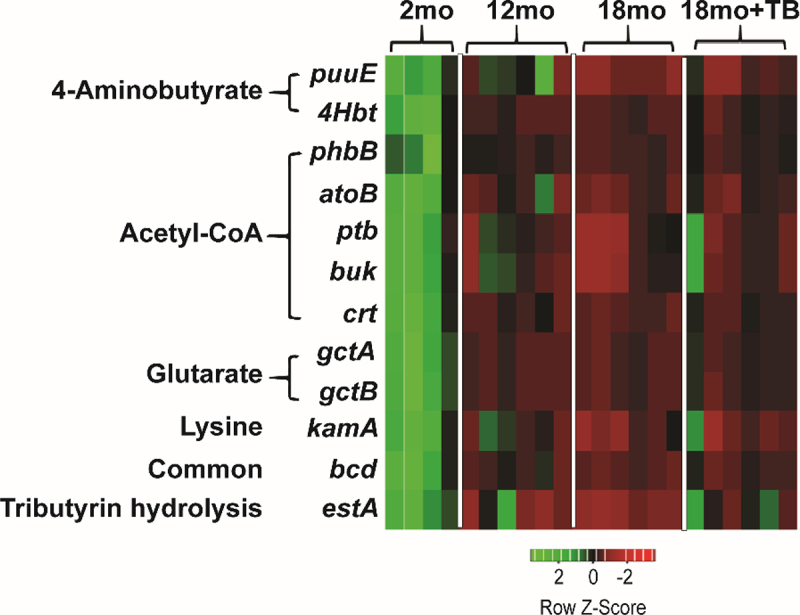
Alterations in the abundance of butyrate-synthesizing genes observed in 3×Tg mice.

The effects of age and TB supplementation on the functional characteristics of the microbiome associated with butyrate synthesis were examined through inferred metagenomics. Putative determination of butyrate-synthesizing genes was achieved from PICRUSt2 output using in-house updated inventory of butyrate-related genes previously described.^38^ This predictive analysis showed that the gut microbiome of 12 and 18 month 3×Tg mice had a significant reduction in the abundance of butyrate-synthesizing genes associated with the four-butyrate synthesizing pathways, namely, acetyl-CoA, lysine, 4-aminobutyrate, and glutarate pathways (Table 1, Figure 3). Furthermore, among the four pathways, genes involved in the acetyl-CoA butyrate synthesizing pathway were most affected with aging in 3×Tg mice. Notably, oral TB supplementation mitigated age-associated decreases in butyrate synthesizing genes (Table 1, Figure 3). Additionally, the gene encoding the putative TB esterase (*estA*), which hydrolyzes TB to generate butyrate, was predicted to be significantly reduced in 12 and 18 month 3×Tg mice compared to 2 months old, indicating a potentially decreased capacity of the gut microbiome to derive butyrate from dietary TB in aging mice. Accordingly, the expression of *estA* was predicted to be increased in 18 month old mice treated with TB compared to those without treatment (*p* = 0.030; Table 1).

Heat maps were generated for 12 distinct butyrate synthesis associated genes as identified by PICRUSt2 analysis for 2, 12, 18 and 18 month+TB groups. The heat maps show genes from all four butyrate biosynthetic pathways, 4-aminobutyrate, acetyl-CoA, glutarate, lysine, and common pathways, as well as the putative tributyrin esterase that hydrolyzes TB to produce butyrate. The red color denotes the genes that are present in low abundance or absent and the green color denotes the genes that are highly abundant. *puuE*: 4-aminobutyrate transaminase, 4Hbt: butyryl-CoA:4-hydroxybutyrate CoA transferase, *phbB*: acetoacetyl-CoA reductase, *atoB*: acetyl-CoA acetyltransferase, *ptb*: phosphate butyryltransferase, *buk*: butyrate kinase, *crt*: crotonase, *gctA*: glutaconate CoA transferase (α subunit), g*ctB*: glutaconate CoA transferase (β), g*ctA*: glutaconyl-CoA decarboxylase (α, β subunits), *kamA*: lysine-2,3-aminomutase, *bcd*: butyryl-CoA dehydrogenase (including electron transfer protein α, β subunits, ter1-trans-2-enoyl-CoA reductase (NAD+)), *estA*: putative tributyrin esterase.

**Table 1.** Effects of aging and TB supplementation on the butyrate synthesizing potential of gut microbiota in 3×Tg mice.

Gene	Enzyme name	Butyrate Synthesis Pathway	2mo vs. 12mo		2mo vs. 18mo		12mo vs. 18mo		18mo vs. 18mo+TB	
			Trend	*p*-value	Trend	*p*-value	Trend	*p*-value	Trend	*p*-value
*puuE* or GABA-T	4-aminobutyrate transaminase	4-Aminobutyrate	↓	n.s.	↓	0.023	↓	n.s.	↑	n.s.
4Hbt, *cat2*, or *abfT*	butyryl-CoA:4-hydroxybutyrate CoA transferase	Acetyl-CoA	↓	0.055	↓	0.052	↓	n.s.	↑	0.070
*phbB*	acetoacetyl-CoA reductase	Acetyl-CoA	↓	n.s.	↓	n.s.	↓	0.012	↑	0.049
*atoB*	thiolase	Acetyl-CoA	↓	0.039	↓	0.032	↓	n.s.	↑	n.s.
*ptb*	phosphate butyryltransferase	Acetyl-CoA	↓	n.s.	↓	0.051	↓	n.s.	↑	n.s.
*buk*	butyrate kinase	Acetyl-CoA	↓	0.062	↓	0.037	↓	n.s.	↑	n.s.
*crt*	crotonase	Acetyl-CoA	↓	0.045	↓	0.039	↓	n.s.	↑	0.061
*gctA*	glutaconate CoA transferase α	Glutarate	↓	0.041	↓	0.042	↓	n.s.	↑	n.s.
*gctB*	glutaconate CoA transferase β	Glutarate	↓	0.037	↓	0.038	↓	n.s.	↑	n.s.
*kamA*	lysine-2.3-aminomutase	Lysine	↓	0.049	↓	0.031	↓	n.s.	↑	n.s.
*bcd*	butyrate-CoA dehydrogenase (including electron transfer protein α, β subunits)	Common to all	↓	0.047	↓	0.039	↓	n.s.	↑	n.s.
*estA*	esterase	putative tributyrin esterase	↓	0.036	↓	0.024	↓	n.s.	↑	0.030

Results from Kruskal–Wallis test comparing the abundance of butyrate synthesizing genes in cecal samples of 3×Tg mice at 2, 12 and 18 months of age. TB: tributyrin; n.s.: no significance. Enzyme abbreviations defined in legend for Figure 3.

Collectively, these results demonstrate that a significant component of age-associated dysbiosis in 3×Tg mice involves a decrease in butyrate-producing bacteria which can be treated by TB supplementation.

### 3.4. Oral supplementation with TB significantly reduces both inflammation and oxidative stress markers and prevents hyperphosphorylation of tau

**Figure 4.**
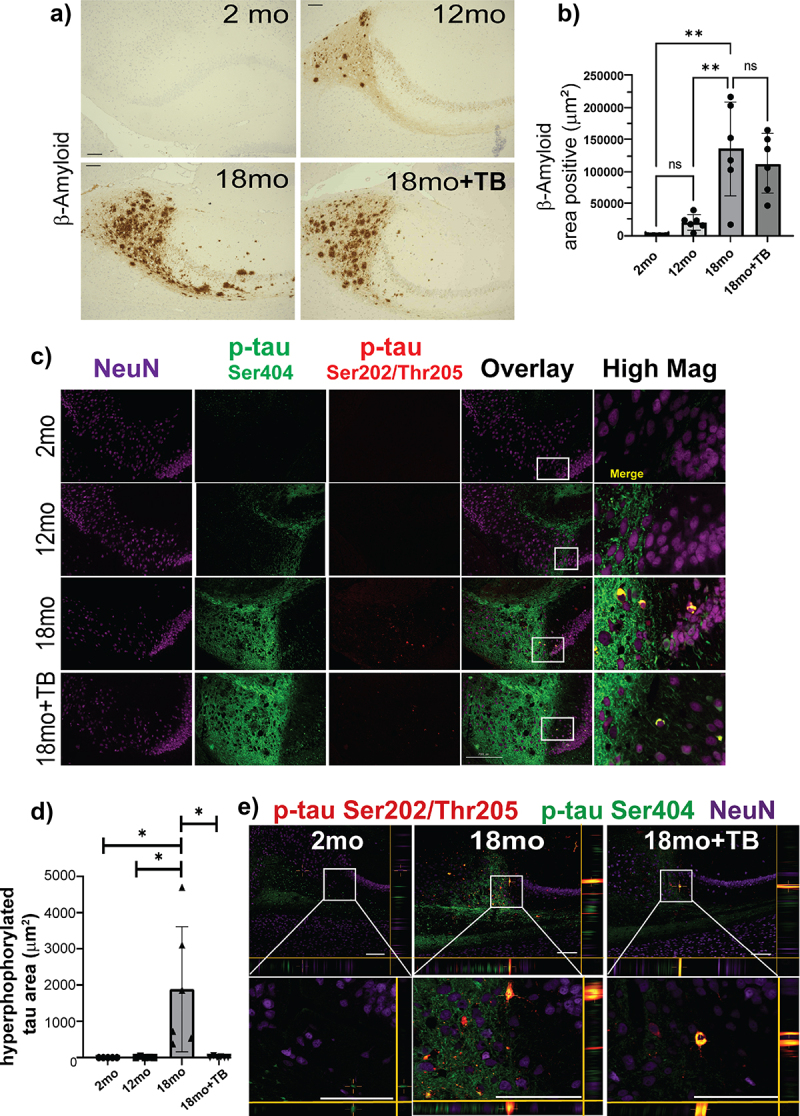
TB prevents hyper-phosphorylation of tau, a critical component of ad-like pathology in 3×Tg mice.

We initially examined the AD-related accumulation of Aβ plaques and phosphorylated tau protein (p-tau), which are linked with the development of neuroinflammation and oxidative stress. As previously reported 3×Tg mice developed AD related accumulation of both Aβ plaques and p-tau in an age-dependent manner (Figure 4). Furthermore, tau phosphorylation states were assessed by i) single staining with D2Z4G (S404) or AT8 (S202, T205) monoclonal antibodies and ii) co-staining and colocalization with D2Z4G and AT8 to indicate hyperphosphorylation. These data showed the accumulation of singly stained p-tau was detected at 12 months of age and significantly increased by 18 months. However, detection of hyperphosphorylated tau, which is indicative of its neuropathic confirmational change, was observed to occur only at an advanced age of 18 months. These Aβ and p-tau changes were predominantly observed in the most inferior component of the hippocampus, the subiculum (Figure 4a,c), which has been linked to spatial and working memory.^39,40^

The presence of Aβ (Aβ40 or Aβ42) in the subiculum of sagittal brain sections was assessed by immunohistochemical staining by D12B2 mAb visualized using DAB. Representative images taken at 20× magnification (a) and area positive quantification using ImageJ (RRID:SCR_003070) are shown (b). To assess tau hyper-phosphorylation, sagittal brain sections were triple stained with two different p-tau mAb, D2Z4G-FITC (p-tauSer404, green), AT8-TRITC (p-tauSer202/Thr205, red) and anti-neuron mAb (NeuN-Cy5; purple). Representative images are shown (c). The images labeled as Overlay + NeuN and High Mag are merged fluorescent images with co-localization of the p-tau stains indicated in yellow. The area shown in the High Mag images is indicated in white boxes in the Overlay + NeuN images and is increased 4-fold. Area positive staining was determined for p-tau Ser404 and p-tau Ser202/Thr205 co-localization (d). The size bar = 200 µm. Bar graphs and error bars represent mean scores ± SD, and data from individual mice are shown as closed markers. Statistics were calculated using parametric ordinary one-way ANOVA with multiple comparisons, and significance indicated as *p < 0.05, **p < 0.01, ***p < 0.001, ****p < 0.0001. Representative confocal microscopy images of fluorescent staining demonstrating co-localization of p-tau stained, AT8 (red; Ser202/Thr205) and D2Z4G (green; Ser404), were significantly increased within subiculum NeuN-positive (purple) neurons of 3×Tg mice by 18 months e). XZ and YZ focal planes are shown to conclusively demonstrate co-localization of these two stains. Co-localized staining shown as yellow was reduced in 18mo+TB. White boxes in the top images are magnified in the lower boxes. Magnification bars = 100 μm.

**Figure 5.**
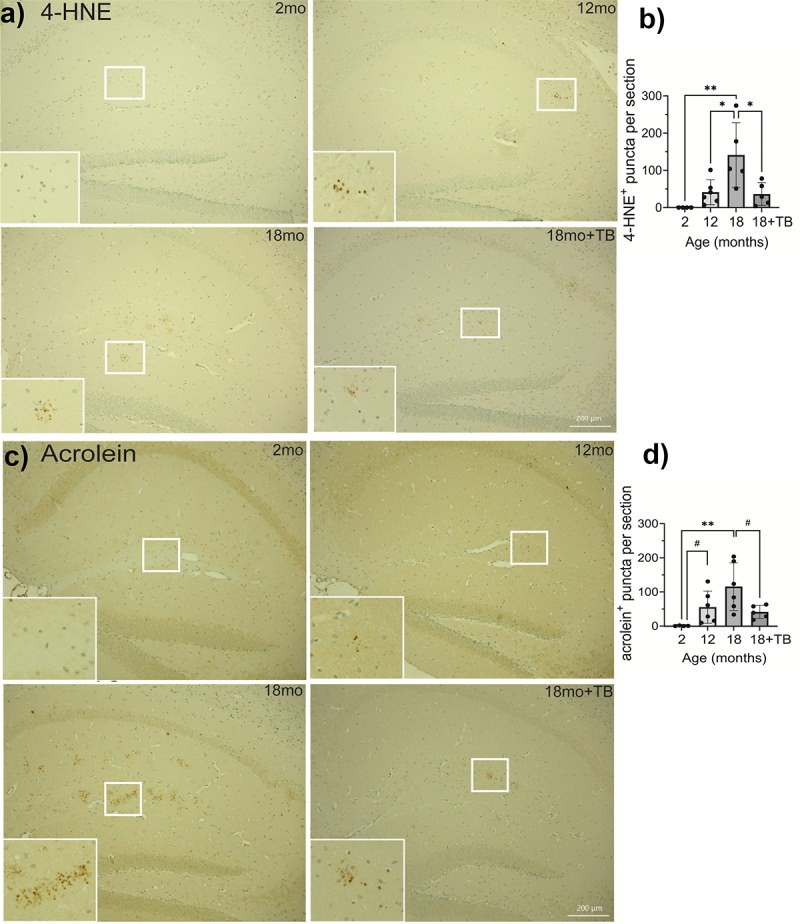
Age-associated oxidative stress is abrogated in 3×Tg mice through oral TB administration.

AD pathology in both human and mouse models, including 3×Tg mice, is characterized by increased oxidative stress which can lead to neuronal damage and death.^41,42^ Notably, both 4-hydroxy-2-nonenal (4-HNE; Figure 5a,b) and acrolein adducts (Figure 5c,d) were increased with age. Little to no 4-HNE or acrolein was detected in the hippocampus at 2 months of age, but both were increased at 12 months, and significantly increased at 18 months of age in the stratum lucidum and the molecular layer of in the hippocampus. The staining for both acrolein and 4-HNE did not appear to be present in the CA regions or in the neurons in the dentate gyrus. Neither marker was significantly increased in the cerebellum or the frontal cortex (data not shown).

Representative images of IHC staining for 4-HNE a) or acrolein adducts c) in sagittal brain sections in the hippocampus were visualized with DAB to assess oxidative stress. Quantification of individual puncta in 4-HNE b) or acrolein adducts d) stained sections was counted using Keyence Analyzer software as described in the Methods section. The majority of positive staining events was localized in the stratum lucidum and the molecular layer of the hippocampus. Magnification bars = 200 µm (a and c, 18mo+TB, white bars on bottom right). Bar graphs and error bars (b and d) represent mean scores ± SD, and data from individual mice are shown in closed circles. Statistics were calculated using parametric ordinary one-way ANOVA with multiple comparisons, and significance is indicated as *p < 0.05, **p < 0.01, or ***p < 0.001. Pairwise comparisons were calculated using unpaired t tests with the Welch’s correction, which does not assume equal SD, with significance indicated as ^#^*p*<0.05.

Supplementation with TB starting at 6-months of age significantly reduced the buildup of both 4-HNE (Figure 5a,b) and acrolein (Figure 5c,d) in the hippocampus of 18 month old animals. Interestingly, although no difference was observed in the accumulation of either Aβ plaques or singly stained p-tau in TB-treated animals (Figure 4), pathological hyperphosphorylation as demonstrated by co-staining and colocalization was markedly inhibited by oral administration of TB (Figure 4c,d). Additionally, confocal imaging analysis of the subiculum of 18 month and TB-treated 18 month mice established that hyperphosphorylation of tau occurred in neurons and was significantly diminished with by oral administration of TB (Figure 4e).

### 3.5. Oral supplementation with TB prevents the age-dependent decrease of histone H3-K9/K14-ac in the hippocampus

**Figure 6.**
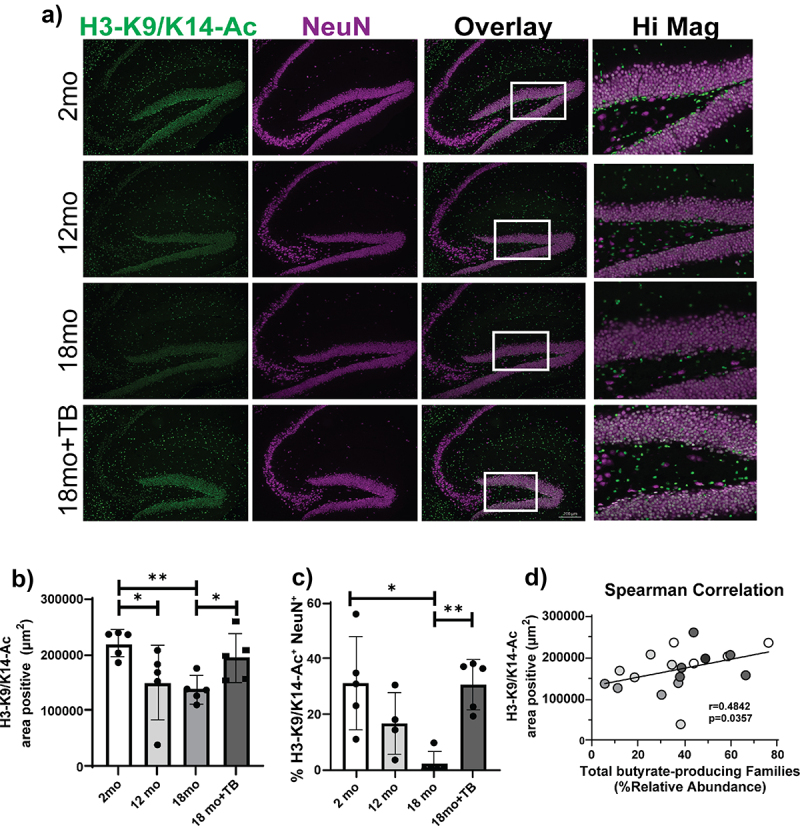
TB supplementation prevents the age-related decrease of H3K9/K14-ac in 3×Tg mice.

An important biological function of butyrate is inhibition of histone deacetylases (HDACs) affecting histone acetylation status and epigenetic regulatory mechanisms.^43^ To determine the functional effects in the CNS exerted by the loss of butyrate-producing bacteria, we assessed the acetylation status of histone 3 on lysine 9 and lysine 14 (H3-K9/K14-Ac) that represents histone modifications associated with active gene regulatory elements.^44,45^ Notably, total H3-K9/K14-Ac declined in the hippocampus with age in 3×Tg brains (Figure 6a,b). These age-associated histone acetylation changes were detected throughout the hippocampus and were predominant among hippocampal neurons present in the dentate gyrus, which are critical for memory formation.^46^ Specifically, dentate gyrus neurons demonstrated robust H3-K9/K14-Ac staining at 2 months that trended lower at 12 months and was significantly decreased at 18 months of age (Figure 6a,b). Importantly, the proportion of NeuN^+^ neurons that stained positive for H3-K9/K14-Ac in the dentate gyrus was completely preserved by TB treatment (18mo+TB group; Figure 6a,c). Notably, a significant direct correlation (*r* = 0.4842, *p* = 0.0357) was observed between total butyrate-producing bacteria and histone H3 acetylation status (H3-K9/K14-Ac) of the hippocampal region (Figure 6d).

Representative images of K9/K14 acetylation of histone 3 in the hippocampus of indicated 3×Tg mice were assessed by immunofluorescent staining using anti-H3K9/K14Ac-FITC (green) and anti-NeuN-Cy5 (purple) antibodies. Double positive cells are shown (white) in the Overlay and Hi Mag panels a). Quantification of total H3K9/K14-Ac staining indicated as area positive staining b). The percent of neurons positive for H3K9/K14-Ac in the dentate gyrus was determined as area of H3K9/K14Ac-FITC and NeuN-Cy5 co-localization divided by the total area of NeuN-Cy5 staining c). Magnification bars = 200 μm (A, Overlay 18mo+TB, white bars on bottom right). Bar graphs and error bars represent mean scores ± SD, and data from individual mice are shown as closed circles. Statistics were calculated using non-parametric Kruskal–Wallis test for multiple comparisons, and Mann-Whitney test for comparing 18 month with and without TB, *p < 0.05, **p < 0.01. Non-parametric Spearman Correlation analysis plot demonstrates the linear association between % relative abundance of total butyrate-producing bacteria with H3K9/K14-Ac area positive staining d). Results from individual mice from all groups of 3×Tg are denoted by different colored circles (white: 2 months old; light gray: 12 months old; medium gray: 18 months old; and dark gray: TB-treated 18 months old). The Spearman correlation coefficient *r* = 0.4842 with *P* value significance *p* = 0.0357.

#### 3.6.1. Tributyrin supplementation abrogates age-dependent short-term and spatial memory deficits in 3×Tg mice

**Figure 7.**
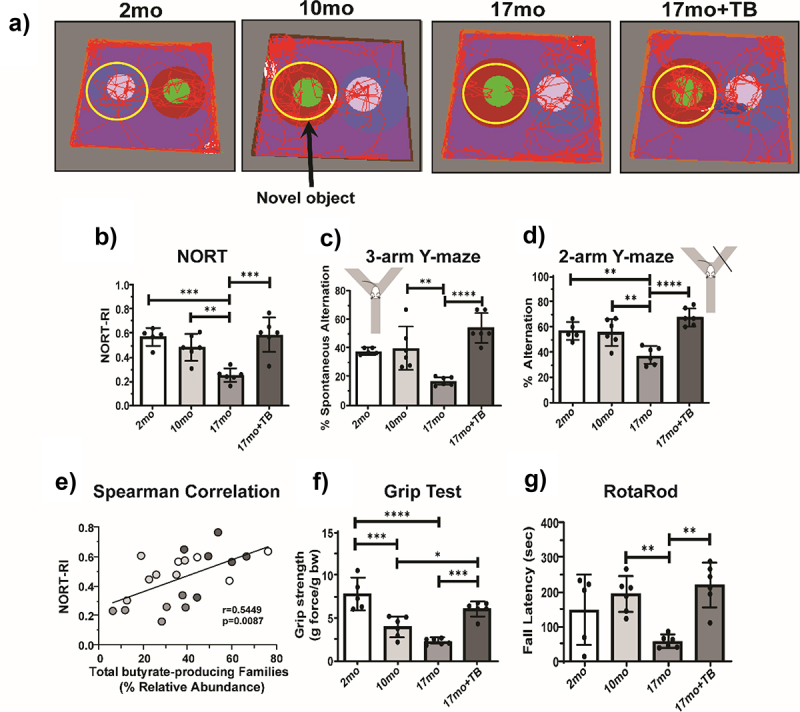
TB protects 3×Tg mice from developing memory and neuromuscular deficits at 17 months.

Short-term episodic memory and spatial memory were measured in 3×Tg mice at 2, 10, and 17 months of age using the Novel Object Recognition Test (NORT) and the Y-maze test, respectively.^30,47^ The NORT and Y-maze tests both exploit rodent instinct to explore new objects and situations^29,48^ to measure hippocampus-associated memory formation and are often used to test for AD-like cognitive deficits in mice.^49^ As shown with representative path tracings, novel object exploration was diminished with age and largely restored in the aged mice given TB (Figure 7a). The NORT recognition index (RI; Figure 7b) indicated preferential interaction with a novel object in a habituated arena four hours after learning a set of two identical objects. In 17 month old 3×Tg mice, the NORT RI was significantly decreased compared to either 2 or 10 month old mice (Figure 7b), indicating deficits in short-term memory. Performance of TB-treated 17 month old mice was similar to that seen in 2 or 10 month old mice. Hippocampal-dependent spatial memory was tested using the Y-maze test, and 17 month 3×Tg exhibited decreased performance in both 3- and 2-arm Y-maze tests compared to 10 month old mice (Figure 7c,d). Oral TB supplementation resulted in significantly better performances in both NORT and Y-maze measurements in 17 month old mice with scores comparable to those of the 10 month 3×Tg mice (Figure 7a–c). Moreover, a strong positive correlation (*r* = 0.5449, *p* = 0.0087) between total butyrate-producing bacteria and NORT RI was observed (Figure 7e). Taken together, these data suggest that age-associated losses in butyrate-producing communities of the gut microbiome played a significant role in the development of short-term memory deficits in 3×Tg mice

Schematic depictions of representative travel traces captured during the NORT novel object probe stage four hours after the familiar object learning stage from the indicated groups of 3×Tg a). The yellow circles indicate placement of the novel object and its surrounding area, and the red lines trace the route of travel for a representative mouse during the 5 minute recording. Calculated NORT recognition indexes (RIs) indicating a lost preference in 17 month old (17mo) 3×Tg mice for the novel object, while 17 month old +TB (17mo+TB) treated mice retained novel object recognition b). The percentage of spontaneous alternations observed in the 3-arm Y-maze trial c) and the percentage of alterations in the 2-arm Y-maze trial d) are also shown. Non-parametric Spearman Correlation analysis plot demonstrates the linear association between % relative abundance of total butyrate-producing bacteria with NORT-RI e). Results from individual mice from all groups of 3×Tg are denoted by different colored circles (white: 2 months old; light gray: 12 months old; medium gray: 17 months old; and dark gray: 17 months old +TB). Spearman correlation coefficient was *r* = 0.5449, and the *P* value indicates significance at *p* = 0.0087. Neuromuscular function in 3×Tg mice was measured with the fore limb Grip Test, measuring the amount of force at which mice lose their grip on a wire mesh, and is depicted as grip strength f). The time to fall or latency when placed on an accelerating RotaRod apparatus was measured in seconds for 3×Tg mice g). Bar graphs and error bars represent mean scores ± SD, and data from individual mice are shown in closed circles. Statistics were calculated using ordinary one-way ANOVA with Tukey post-hoc comparisons test with single variance and are indicated as *p < 0.05, **p < 0.01, ***p < 0.001, ****p < 0.0001.

.

#### 3.6.2. Tributyrin supplementation effectively treats losses in neuromuscular function and motor coordination in 3×Tg mice

As shown in other studies of AD mouse models expressing tau polymorphic proteins,^50,51^ both muscle function and motor coordination declined with age in the 3×Tg mice (Figure 7f,g). Although fore limb grip strength (gram force/body weight) was decreased by 10 months with no further decline at 17 months, TB treatment protected against this loss of strength (Figure 7f). Balance and coordination performance on the rotarod test varied widely in both the 2 and 10 month 3×Tg mice, but at 17 months they exhibited markedly shortened latency to fall time, indicative of decreased motor coordination and/or balance. The TB-treated 17 month old mice, however, exhibited motor coordination comparable to 10 month old mice and significantly higher than that of untreated 17 month old mice (Figure 7g).

These studies demonstrated that TB treatment beginning at 6 months of age dramatically inhibited the loss of neurologic function in 3×Tg mice, as indicated by increases in memory, cognition, and neuromuscular function. Therefore, TB prevented the progression of clinical signs of cognitive impairment in the context of AD-like pathogenesis.

## 4. Discussion

Pre-clinical and clinical studies have documented the incidence of decreased proportions of butyrate-producing bacteria in association with AD; however, information regarding the dynamic changes in these populations during the development of AD remains largely undetermined.^52^ Accordingly, we sought to comprehensively examine the age-associated, temporal decline in butyrate-producing bacterial communities that correlate with the development of neuropathological changes in the 3×Tg mice. The initial taxonomic assessment of the age-associated changes in the gut microbiome demonstrated a significant decrease in the phylum Firmicutes. Notably, the Firmicutes phylum encompasses the majority of butyrate-producing bacteria within distinct bacterial families. Our study leveraged the well-substantiated compendium of butyrate-producing communities^38^ and identified five butyrate-producing families belonging to the Firmicutes phylum, that collectively demonstrated an age-associated decline in their relative abundance and a concurrent reduction in cecal butyrate production. Further, this analysis also revealed that among these downward trending families, there was a significant age-associated decrease in *Ruminococcaceae*, one of the largest butyrate-producing bacterial families. Cross-sectional clinical studies in AD patients have also reported decreases in abundance of Firmicutes along with decline in *Ruminococcaceae* and *Lachnospiraceae* families.^53–57^ This supports the clinical relevance of the findings in the pre-clinical 3×Tg animal model and comprehensively demonstrates the longitudinal, age-associated decrease in butyrate-producing families in the context of AD pathogenesis. It is noteworthy that the nTg mice that are devoid of any AD-related neuropathology did not demonstrate longitudinal, age-associated changes in microbial diversity or in total butyrate producing bacteria. With regards to age-related changes in the gut microbiome, cross sectional analysis has demonstrated a decrease in butyrate-producing bacteria in relatively older (≥20 months) C57BL/6J mice.^58^ Hence, it is conceivable that the nTg mice could potentially experience a decrease in butyrate-producing bacteria at a relatively later age (beyond the 17-month study time point). These data demonstrate that there was a distinct difference between 3×Tg mice and their nTg counterparts in the development and trajectory of age-related gut microbial dysbiosis marked by loss of total butyrate-producing bacterial communities. Moreover, these data also imply that the temporal decrease in butyrate-producing bacteria that occurs in 3×Tg mice at a relatively earlier age (observable at 12 months) prior to the development of neuropathological changes, is likely a consequence of the combinatorial effects of both aging and Alzheimer’s disease phenotype.

In addition to taxonomic analyses showing compositional changes in butyrate-producing bacteria, inferred metagenomics analysis provided information regarding the functional alteration in butyrate metabolic pathways associated with the butyrate-producing microbial communities. Earlier work has demonstrated that in the context of butyrate-producing microbial communities, targeting complete pathways is a more compelling way to predict the butyrogenic function.^38,59^ Accordingly, our metagenomic analysis of the overall butyrogenic function of the gut microbiome identified the acetyl-CoA pathway as the main bacterial pathway for butyrate production that was significantly decreased in an age-dependent manner in the 3×Tg mice. The temporal age-associated effects on the reduction in the acetyl-CoA pathway is a highly significant and relevant component of the loss of butyrate production and functional dysbiosis of the microbiome. It is noteworthy that the murine microbiome shares similarities with the human microbiome which also comprises acetyl-CoA as the main butyrate synthesizing pathway harbored by the majority of butyrate-producing bacteria that drives butyrate production.^38,59^ Importantly, these data in 3×Tg mice demonstrate that the observed temporal decrease in age-associated butyrate-synthesizing bacteria: i) is a major component of the loss of overall diversity that progressively declines over time, ii) corresponds with the functional loss of butyrate synthesis as indicated by the decrease in cecal butyrate levels, and iii) correlates with the development of AD-related neuropathological changes. It is noteworthy, that the gut microbial dysbiosis evident at 12 months preceded the manifestation of AD-related neuropathology at 18 months including oxidative stress, tauopathy (hyperphosphorylation of tau), and memory deficits as well as loss of muscle strength and development of neuromuscular dysfunction. Importantly, these temporal changes in the gut microbiome were indicative of its potential causal role in the onset and progression of AD pathology.

The causal relationship of age-associated compositional and functional decline in the butyrate-producing bacteria in AD pathogenesis was corroborated by the outcomes of the oral administration of the butyrate prodrug, tributyrin. TB administration was purposely initiated at 6 months of age in 3×Tg mice, which corresponds to early-stage development of progressive amyloid and tau pathologies.^11^ Importantly, TB administration prevented the age-related decline in total butyrate-producing families and cecal butyrate levels that significantly correlated with the attenuation of neuropathological and cognitive changes associated with AD pathogenesis. However, the observed beneficial prophylactic effect of TB was independent of β-amyloid levels and senile plaque formation, which remained unaffected. These data are in accordance with the observations in a related transgenic mouse model of amyloid pathology and cognitive deficits, wherein reducing endogenous tau levels prevented behavioral deficits without altering their high Aβ levels.^60^ Moreover, in clinical studies, the relevance of poor correlation between brain amyloid plaque burden and disease frequency, severity, or neuronal loss has been recognized.^61,62^ Further, identification of brain amyloidosis in about 30 to 40% of individuals in their 70s and 50% of centenarians without cognitive impairment has been observed.^63,64^ Taken together, our data along with earlier work strongly supports the key role of hyperphosphorylated tau in AD pathogenesis in the 3×Tg animal model of AD which can be prevented by oral administration of TB.

After oral administration, TB is hydrolyzed to butyrate, increases plasma butyrate levels, and provides HDAC inhibitory capabilities.^16,17^ Commensurate with the biological function of HDAC inhibition, TB/butyrate prevented the extensive age-associated temporal decline in H3K9- and H3K14-acetylation levels in hippocampal CA1 and CA3 neuronal populations and preserved memory function. Histone H3 acetylation at both lysine 9 and 14 (H3K9/K14-Ac) is well-established as histone modifications that characterize a transcriptionally active promoter chromatin state and is often associated with ongoing transcription.^65,66^ Hence, the temporal decrease in H3K9/K14 acetylation levels potentially represents transcriptional repression of relevant genes that contribute to memory function. In this regard, earlier work has demonstrated that a decrease in overall brain histone acetylation in Tg2576 AD transgenic mice leads to transcriptional downregulation and expression of genes involved in synaptic integrity and plasticity, leading to the impairment of memory function.^67^ Moreover, in different animal models of AD, inhibition of HDAC function by the application of pan and isoform selective HDAC inhibitors, including butyrate, have been observed to counteract deficits in memory and learning associated with AD pathogenesis.^68,69^ The present data strongly suggest that in 3×Tg mice, age-associated antecedent decreases in butyrate-producing communities and butyrate synthesis plays a causal role in the deregulation of hippocampal HDACs and ensuing deacetylation of specific histone lysine residues, likely leading to transcriptional repression of genes related to memory and learning. The identity of those specific genes affected by loss of butyrate-producing bacteria and butyrate levels that could affect memory function remain to be defined.

The beneficial effects of TB also suppressed tau hyperphosphorylation, a hallmark of AD pathology. This neuroprotective effect of TB could potentially have been elicited via its HDAC inhibitory function and involving the inhibition of glycogen synthase kinase 3β (GSK3β). HDAC inhibitors are known to inhibit GSK3β, a kinase that participates in the pathogenic hyperphosphorylation of tau during AD pathogenesis.^66,69–71^ Additionally, a selective inhibitor of HDAC3 has also been shown to decrease tau hyperphosphorylation by directly inhibiting HDAC3-dependent mechanisms that mediate tau phosphorylation.^72^

Besides preventing the development of AD-associated neuropathology and cognitive dysfunction, TB/butyrate also significantly inhibited the accompanying loss of neuromuscular function and motor coordination. Sarcopenia-associated phenotypes involving pathological changes in muscle strength and function along with cognitive decline are observed in human AD patients.^73,74^ It is noteworthy that HDACs play key regulatory roles in skeletal muscle metabolism and have been linked to the development of muscle atrophy and dysfunction.^75^ In view of this, the data suggest that the gut microbial dysbiosis initiated temporal changes in HDACs in skeletal muscle, leading to sarcopenia-related phenotypes that are therapeutically targeted by the HDAC-inhibitory function of TB/butyrate. Indeed, butyrate, via its HDAC inhibitory function, has been shown to attenuate age-related sarcopenia involving the loss of skeletal muscle mass and function.^76^

The neuroprotective effects of oral administration of TB also involved antioxidant function which was evident from the inhibition of the age-associated formation and accumulation of the major lipid oxidation by-products, 4-HNE and acrolein. Earlier work has demonstrated that lipid peroxidation by-products are clinically relevant biomarkers of oxidative stress and contribute significantly to the development of the pathophysiology of AD.^77^ With regards to the relationship to other pathological features of AD, the lipid peroxidation product acrolein is known to instigate tau hyperphosphorylation through the activation of stress activated protein kinases like GSK3β and p38 kinase.^77–80^ The inhibition of the age-associated formation of neuropathologic lipid peroxidation byproducts 4-HNE and acrolein by TB/butyrate is likely mediated by the induction of glutathione/glutathione-S-transferase (GST) antioxidant system.^81–83,84^

Overall, these data support our assertion that the age-associated decline in butyrate-producing bacteria in the gut microbiome acts as a key pathogenic feature of the microbiome-gut-brain axis during the onset and progression of AD. Moreover, the data demonstrate that regulation of butyrate-producing bacteria and consequent butyrate synthesis could be a significant therapeutic treatment for the progression of AD.

## Supplementary Material

Supplemental Material

## Data Availability

The data that support the findings of this study are available from the corresponding author, [SSB], upon reasonable request in compliance with NIH data sharing guidelines.

## Abbreviations

3×Tg: 3×Tg-AD mouse model
4-HNE: 4-hydroxy-2-nonenal
acetyl-CoA: acetyl-coenzyme A
AD: Alzheimer’s disease
Aβ: amyloid-β
h: hours
NFT: neurofibrillary tangle
nTg: non-transgenic
PBS: phosphate buffered saline
PICRUSt2: Phylogenetic investigation of communities by reconstruction of unobserved states 2
p-tau: phosphorylated tau protein
SCFA: short-chain fatty acids
TB: tributyrin
