# Supplementary material for: Age-associated temporal decline in butyrate-producing bacteria plays a key pathogenic role in the onset and progression of neuropathology and memory deficits in 3×Tg-AD mice
Source: Gut Microbes. 2024 Aug 25;16(1):2389319. doi: 10.1080/19490976.2024.2389319 (PMC11346541; doi:10.1080/19490976.2024.2389319)
Supplement: Supplemental Material [file KGMI_A_2389319_SM2519.zip › Supplementary Table S1.docx]

**Supplementary Table 1**: Details on primary antibodies used for immunohistochemistry or immunofluorescent staining

| Antibody specificity | Host  (clone) | Dilution used for stain | Supplier  (catalog no.) | Research Resource Identifier |
| --- | --- | --- | --- | --- |
| 4-HNE | Rabbit (polyclonal) | 1:100 | Alpha Diagnostic Intl (HNE11-S) | RRID:AB_2629282 |
| Acrolein | Rabbit (polyclonal) | 1:500 | Cell Sciences (PA2049) | RRID:AB_170939 |
| Amyloid β | Rabbit (D12B2) | 1:400 | Cell Signaling Technology (9888) | RRID:AB_2797713 |
| p-tau (Ser404) | Rabbit (D2Z4G) | 1:200 | Cell Signaling Technology (20194) | RRID:AB_2798837 |
| p-tau (Ser202, Thr205) | Mouse (AT8) | 1:200 | ThermoFisher Scientific (MN1020) | RRID:AB_223647 |
| H3K9/K14-Ac  double acetylation | Rabbit (polyclonal) | 1:200 | Cell Signaling Technology (9677) | RRID:AB_1147653 |
| NeuN | Guinea Pig, (polyclonal) | 1:200 | MilliporeSigma (ABN90P) | RRID:AB_2341095 |
