# Supplementary figures and images for: Age-associated temporal decline in butyrate-producing bacteria plays a key pathogenic role in the onset and progression of neuropathology and memory deficits in 3×Tg-AD mice
Source: Gut Microbes. 2024 Aug 25;16(1):2389319. doi: 10.1080/19490976.2024.2389319 (PMC11346541; doi:10.1080/19490976.2024.2389319)

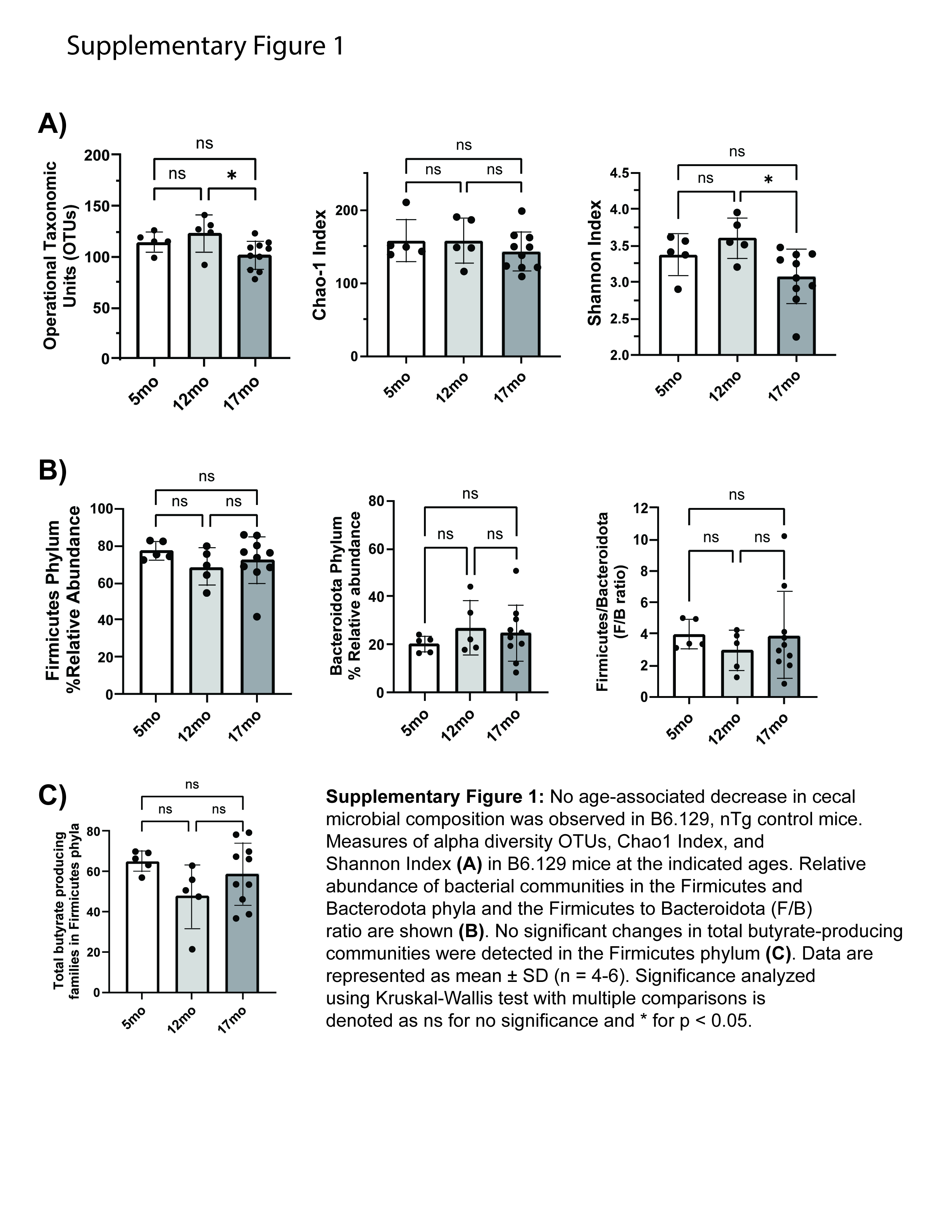

Supplement: Supplemental Material [file KGMI_A_2389319_SM2519.zip › SupFig1.tif]

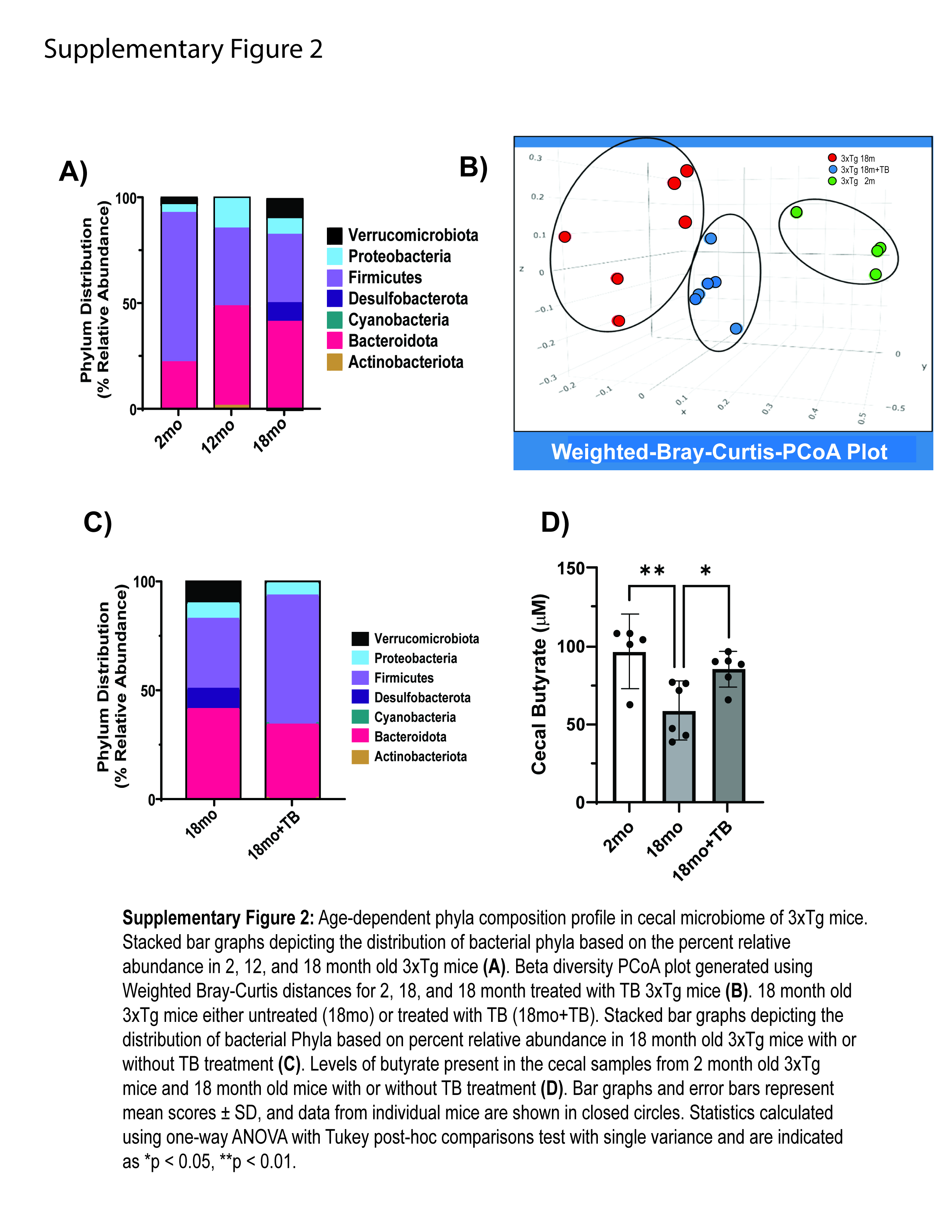

Supplement: Supplemental Material [file KGMI_A_2389319_SM2519.zip › SupFig2.tif]

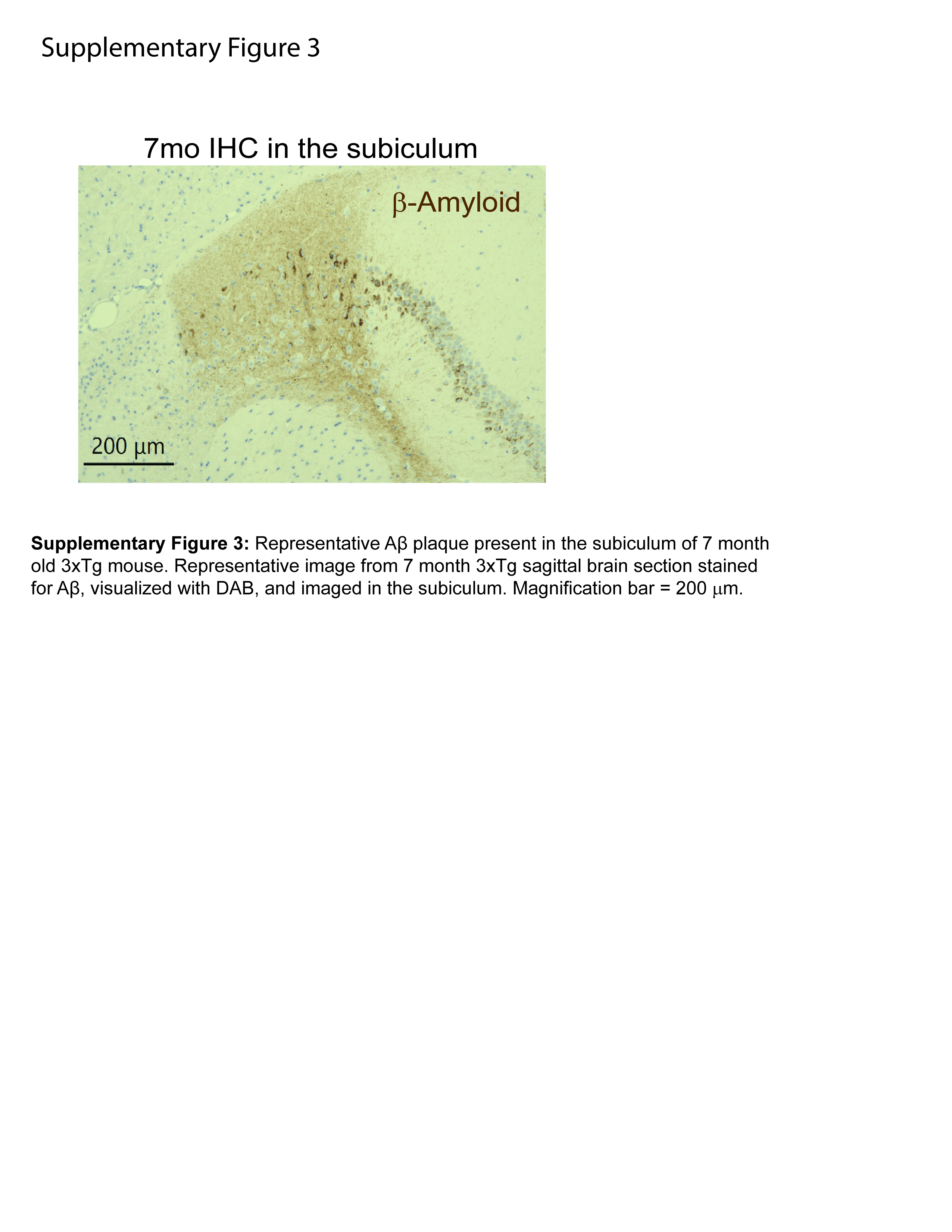

Supplement: Supplemental Material [file KGMI_A_2389319_SM2519.zip › SupFig3.tif]
